# Neuropeptide GPCRs in Neuroendocrinology

**DOI:** 10.3389/fendo.2014.00041

**Published:** 2014-04-04

**Authors:** Hubert Vaudry, Jae Young Seong

**Affiliations:** ^1^INSERM U982, IRIB, University of Rouen, Mont-Saint-Aignan, France; ^2^Korea University, Seoul, South Korea

**Keywords:** neuroendocrinology, neuropeptides, biologically active peptides, G protein-coupled receptors, seven-transmembrane domain receptors, heptahelical receptors, signaling mechanisms, transduction pathways

G protein-coupled receptors (GPCRs) represent the single largest family of plasma membrane receptors, encompassing about 860 members in humans. During the last decades, considerable progress has been made regarding the biochemical identification, signaling mechanisms, allosteric modulation, structural characterization, and pathophysiological implication of GPCRs, which are considered as major targets for the development of new therapeutic agents. Thus, in 2012, Robert J. Lefkowitz and Brian K. Kobila shared the Nobel Prize in Chemistry for their seminal contribution on GPCR structures and functions.

Neuropeptides play a pivotal role in chemical communication in the brain and in peripheral organs. To date, more than 100 neuropeptides have been identified and it is established that a vast majority of them act through GPCRs. Neuropeptide GPCRs are involved in the control of a large array of physiological processes including feeding, reproduction, growth, metabolism, stress response, sleep, and a number of behaviors. The aim of this Research Topic is to illustrate the importance of neuropeptide GPCRs in neuroendocrine communication by gathering a bouquet of 72 review papers and original articles from leading scientists in this fast-evolving field.

We are deeply indebted to all authors who have contributed to this Research Topic and to the dedicated reviewers who helped us reaching the highest quality standards. We gratefully acknowledge the valuable support of the Frontiers team and Mrs Catherine Beau for her invaluable help in the processing of manuscripts.

